# Facile Doping and Functionalization of Molybdic Acid into Nanobiochar to Enhance Mercury Ion Removal from Water Systems

**DOI:** 10.3390/nano14221789

**Published:** 2024-11-07

**Authors:** Safe ELdeen M. E. Mahmoud, Tarek M. Abdel-Fattah, Mohamed E. Mahmoud, Eva Díaz

**Affiliations:** 1 Department of Chemical and Environmental Engineering, Faculty of Chemistry, University of Oviedo, Julián Clavería s/n, 33006 Oviedo, Spain; safeeldeenelsayed@aast.edu; 2 Chemical and Petrochemical Engineering Department, College of Engineering and Technology, Arab Academy for Science and Technology and Maritime Transport, Alexandria 21611, Egypt; 3 Applied Research Center Thomas Jefferson National Accelerator, Facility and Department of Molecular Biology and Chemistry, Christopher Newport University, Newport News, VA 23606, USA; fattah@cnu.edu; 4 Faculty of Sciences, Chemistry Department, Alexandria University, Alexandria 21511, Egypt; memahmoud10@alexu.edu.eg

**Keywords:** artichoke leaf nanobiochar, molybdic acid modifier, mercury (II) capture, kinetics–isotherm–thermodynamics modeling evaluations

## Abstract

Functionalized nanomaterials with surface-active groups have garnered significant research interest due to their wide-ranging applications, particularly in water treatment for removing various contaminants. This study focuses on developing a novel, multi-functional nanobiosorbent by synthesizing nanosized biochar from artichoke leaves (NBAL) and molybdic acid (MA). The resulting nanobiosorbent, MA@NBAL, is produced through a microwave-irradiation process, offering a promising material for enhanced environmental remediation. The characteristics of assembled MA@NBAL were evaluated from SEM-EDX, XPS, TGA, FT-IR, and zeta potential detection. The size of particles ranged from 18.7 to 23.7 nm. At the same time, the EDX analysis denoted the existence of several major elements with related percentage values of carbon (52.9%), oxygen (27.6%), molybdenum (8.8%), and nitrogen (4.5%) in the assembled MA@NBAL nanobiosorbent. The effectiveness of MA@NBAL in removing Hg(II) ions was monitored via the batch study method. The optimized maximum removal capacity of Hg(II) ions onto MA@NBAL was established at pH 6.0, 30.0 min equilibrium time, and 20 mg of nanobiosorbent, providing 1444.25 mg/g with a 10.0 mmol/L concentration of Hg(II). Kinetic studies revealed that the adsorption process followed a pseudo-second-order model, with R^2^ values ranging from 0.993 to 0.999 for the two tested Hg(II) concentrations, indicating excellent alignment with the experimental data. This suggests that the chemisorption mechanism involves cation exchange and complex formation. Isotherm model evaluation further confirmed the adsorption mechanism, with the Freundlich model providing the best fit, yielding an R^2^ of 0.962. This result indicates that Hg(II) adsorption onto the surface of MA@NBAL nanobiosorbent occurs on a heterogeneous surface with multilayer formation characteristics. The results of the temperature factor and computation of the thermodynamic parameters referred to endothermic behavior via a nonspontaneous process. Finally, the valid applicability of MA@NBAL nanobiosorbent in the adsorptive recovery of 2.0 and 5.0 µg/mL Hg(II) from contaminated real aquatic matrices was explored in this study, providing 91.2–98.6% removal efficiency.

## 1. Introduction

Heavy metal contamination in water resources, primarily driven by industrial activities, poses a major environmental threat due to the high toxicity and bioaccumulative nature of these pollutants [1]. Among them, mercuric compounds are particularly hazardous, exhibiting toxicological properties that lead to serious health risks. These risks are primarily linked to the inhalation and ingestion of organic mercury from aquatic organisms, resulting in mercury poisoning and associated diseases [2]. Once absorbed, mercury distributes throughout the body’s tissues, leading to bioaccumulation and severe consequences such as kidney damage, blood vessel congestion, and neurotoxicity [3]. Furthermore, mercury and its derivatives are non-biodegradable, persisting in water resources, wastewater, and sediments. Their environmental release triggers complex physical and chemical reactions [4]. Given the significant threats posed to human health, it is essential to reduce mercury pollutants from contaminated sources. This can be achieved through various recovery and removal techniques, which serve as a critical first step in mitigating the impact of mercury pollution [5].

Numerous practical techniques have been employed to recover mercury species from water, focusing on methods such as adsorption, photocatalytic remediation, chemical reduction, electrodialysis, evaporative recovery, reverse osmosis, membrane filtration, and ion exchange [6,7,8]. Among these, adsorption technologies have gained significant attention due to their simplicity, high efficiency, low cost, and reliability [9]. Recently, various low-cost adsorbents, biosorbents, and nanosorbents have been applied to remove mercury ions from wastewater [10]. Biochar is a notable example, widely used for the removal of both organic and inorganic pollutants [11], which are typically prepared from raw materials such as agricultural waste, sewage sludge, and animal feces, via oxygen-free thermal treatment, resulting in fine-grained, porous, carbon-rich materials with various functional groups [12]. Thanks to their high sorption efficiency, pristine biochar materials have been employed as alternative adsorbents for the removal of toxic metal ions and other harmful pollutants from water [13,14,15,16,17,18,19,20].

The design and synthesis of biochar materials with novel functionalities for efficient mercury ion removal from water have gained significant attention in recent years [21]. For example, thiolate-functionalized biochar was developed and studied for mercury speciation transformation, focusing on the influencing factors and interaction mechanisms [22]. Additionally, the effect of hydrothermal pretreatment on biochar derived from corn straw was reported to enhance mercury-removal performance [23]. Another study investigated the removal of mercury and arsenic ions from wastewater using nanoporous biochar-supported-poly(2-aminothiophenol) [24]. Enhanced mercury removal was also demonstrated using chlorine-hierarchically porous biochar, synthesized with CaCO_3_ as a template [25]. Investigations into unmodified and Fe-modified biochars revealed their mercury removal-mechanisms using synchrotron-based methods [26]. A plasma technique for introducing sulfur-containing functional groups into biochar was found to significantly improve mercury removal efficiency [27]. Sulfurized biochar was further studied for its mercury-removal mechanisms from aqueous solutions [28]. Moreover, biochar nanobiosorbents decorated with Mn-ferrite, Zn–Al layered double hydroxides (LDHs), and cellulose were prepared and characterized for effective mercury (II) ion remediation from water [29]. A combined experimental and density functional theory (DFT) approach was used to capture Hg(II) and Cd(II) from aquatic systems using Fe–Mn oxide-modified biochar [30].

Molybdenum ions typically exist in their hexavalent oxidation state, commonly found as compounds like molybdenum trioxide (MoO_3_), sodium molybdate dihydrate (Na_2_MoO_4_·2H_2_O), ammonium dimolybdate ((NH_4_)_2_Mo_2_O_7_), and ammonium heptamolybdate tetrahydrate ((NH_4_)_6_Mo_7_O_24_·4H_2_O) [31,32]. Dissolved Mo(VI) ions generally exist in the form of molybdate (MoO_4_^2^⁻), with their speciation depending on concentration and pH conditions. Additionally, molybdenum can occur as molybdenum disulfide (MoS_2_) and molybdenum dioxide (MoO_2_). Molybdenum compounds are widely used as catalysts and chemical reagents [31,32]. For example, a recent study demonstrated microalgae-mediated bioremediation of molybdenum (V), showing its potential for pollutant removal and biofuel production [33]. A comparative evaluation of two-dimensional MXene and MoS_2_ for Cr(VI) removal from water has also been reported [34]. Moreover, reinforced ionic liquids on magnetic nanoparticles shaped by molybdenum crown polyoxometalates have shown high efficiency in removing both toxic metals and microplastics from water [35]. Furthermore, β-MoO_3_ synthesized via evaporation of nitric acid and molybdic acid was evaluated for its performance in the partial catalytic oxidation of methanol [36].

Mercury pollution has recently been detected in Western Harbour, Alexandria, Egypt, due to the discharge of contaminated effluents into El-Mex Bay. The pollutants primarily originate from a chloro-alkali plant, the petroleum quay at the Noubaria Canal outfall, and other industrial activities in the region [37]. In response, this research aims to design, develop, and produce an innovative, sustainable, low-cost, recyclable, and efficient nanobiosorbent for the removal of mercury ions from polluted wastewater. To achieve this, the investigation focuses on the surface modification of nanobiochar derived from artichoke leaves (NBAL), a low-cost, sustainable material with molybdic acid (H_2_MoO_4_, MA), a common metallic acid. The combination forms a novel nanobiosorbent, MA@NBAL, synthesized via a facile microwave-assisted method. The MA@NBAL was characterized using various techniques to confirm its surface and chemical structures and was further applied as an effective nanobiosorbent for removing Hg(II) ions from water. The removal efficiency of Hg(II) was optimized under batch-mode conditions, considering critical experimental parameters such as pH, MA@NBAL dosage, reaction time (kinetic studies), temperature (thermodynamic parameters), initial Hg(II) concentration (adsorption isotherms), and ionic strength. These factors significantly influence the removal efficiency of diverse pollutants. Additionally, the potential applications of MA@NBAL in removing Hg(II) from wastewater were explored. This study provides valuable insights into the applicability of MA@NBAL nanobiosorbent for the design of a mercury-laden wastewater treatment plant.

## 2. Materials and Methods

### 2.1. Materials

All reagents and chemicals were used as received and purchased, including their specifications. Molybdic acid (H_2_MoO_4_ = MoO_3_·H_2_O, MW = 161.95 and assay ≥ 85.0% MoO_3_ basis), mercuric acetate (Hg CH_3_COO)_2_, MW = 318.68 and assay ≥ 98.0%) and hydrochloric acid (HCl, MW = 36.46 and assay 99.0%), and diphenylthiocarbazone (dithizone, C_6_H_5_NHNHCSN=NC_6_H_5_, MW = 256.33 and assay ≥ 85.0%) were all purchased from Sigma-Aldrich(St. Louis, MO, USA). Sodium chloride (NaCl, MW = 58.44 and assay 98.0%) was purchased from BDH, Poole, England, while sodium hydroxide (NaOH, MW = 39.99 and assay > 99.0%) and ethylenediaminetetraacetate disodium dehydrate (EDTA, C_10_H_14_N_2_Na_2_O_8_·2H_2_O, MW = 372.24 and assay ≥ 99.0%) were collected from Riedel de Haën (AG, Seelze-Hannover, Germany).

The employed instrumentations, including the specifications and operational conditions, are listed in Table S1 (Supplementary Materials).

### 2.2. Facile Microwave-Assisted Doping of Molybdic Acid onto Nanobiochar (MA@NBAL)

The procedure for surface immobilization of molybdic acid (MA) onto the derived nanobiochar from Cynara scolymus nanobiochar (NBAL) was performed according to a combined thermo-hydro-solvo-microwave process by using only ethanol-distilled water-microwave irradiative heating [38]. A total of 1.0 g of molybdic acid was reacted with 5.0 g NBAL (1:5 w) in presence of 3 mL of EtOH and 20 mL of DW. This mixture was subjected to grinding by a mortar and then heated for 3 min in a microwave oven. The formed MA@NBC material was mixed (5 mL DW), heavily ground, and microwave-irradiated for 2 more min. The above steps were triplicated for complete doping of MA onto NBAL surface for MA@NBAL formation.

### 2.3. Sorption Investigation of Hg(II) by MA@NBAL Nanobiosorbent

The adsorptive interaction of Hg(II) using the assembled MA@NBC nanobiosorbent was accomplished using the batch mode. A specific mass of mercuric salt was dissolved in DW to prepare stock Hg(II) solution, from which the desired 5 mmol/L and 10 mmol/L of Hg(II) were prepared. The batch-adsorption procedures were conducted with diverse experimental parameters, including solution pH, shaking time, reaction temperature, MA@NBAL dosage, and ionic strength, in order to identify the optimum conditions [39]. Two different initial Hg(II) concentrations, viz., 5 and 10 mmol/L, were applied to figure out the adsorptive removal capacity (mg/g) according to triplicate experiments.

To evaluate the impact of pH on removal capacity (mg/g) values, an amount of 2.0 g/L of MA@NBAL nanobiosorbent was mixed with 10.0 mL of aqueous solution containing Hg(II) ions as 5 and 10 mmol/L initial concentrations regulated to the required initial pH 2.0–6.0 in contact solutions via addition of the required buffer solution. These mixtures were vibrated for 30.0 min at 250 rpm using a mechanical shaker. The MA@NBAL nanobiosorbent, along with Hg(II) adsorbed, was isolated from solutions via filtration. The remaining Hg(II) was analyzed via EDTA titration to characterize the removed Hg(II) by MA@NBAL nanobiosorbent according to the following Equation (1):

(1) $$q_{e}=(C_{o}-C_{t})\times \frac{V}{W}$$

where *C_o_* and *C_t_* denote the initial and final Hg(II) ions (mg/L) before and after reaction, respectively. *W* is the MA@NBAL mass (mg), and *V* is the volume (L).

The impact of shaking time factor on Hg(II) ion-removal process via MA@NBAL was investigated and monitored by mixing 10.0 mL Hg(II) solution (5 and 10 mmol/L) with 2.0 g/L of nanobiosorbent and controlled to the optimum pH, 6.0. Various shaking times (5–60 min) were explored by using a 250 rpm automatic shaker. Then, the MA@NBAL nanobiosorbent-loaded-Hg(II) ions were isolated from solutions via filtration to identify the capacity value (mg/g) according to Equation (1). The results of time factor were used to evaluate the interaction mechanisms of Hg(II) onto MA@NBAL via the postulates of four diverse kinetic models (*pseudo*-first-order, *pseudo*-second order, intra-particle diffusion, and Elovich).

Optimization of Hg(II)-removal process using different MA@NBAL masses was investigated with batch experiments using 0.5–10 mg/L. In a typical procedure of optimum conditions of pH 6, and adsorption time of 30.0 min, 10.0 mL of different Hg(II) ions (5 and 10 mmol/L) was added to the selected mass, and the capacity values (mg/g) were determined using Equation (1), as explained above.

The contribution of various initial Hg(II) ion concentrations to sorption behavior using MA@NBAL nanobiosorbent were also examined and evaluated by using 5–50 mmol/L. A total of 10.0 mL at pH 6 of each concentration was reacted with 2.0 g/L of MA@NBAL and 30.0 min shaking time, following the steps explained above.

The reaction temperature impact at 25.0–60.0 ± 1.0 °C was used to follow Hg(II) sorption onto MA@NBAL, as well as calculate various thermodynamic parameters. A total of 10 mL of Hg(II) solution (5 and 10 mmol/L at pH 6.0) were combined with 1.0 g/L of MA@NBAL, and all were vibrated using a digital temperature control shaker for 30.0 min. The remaining Hg(II) ion concentrations were detected, as illustrated above, to identify the capacity (mg/g) values. To figure out the affinity of MA@NBAL nanobiosorbent for binding with Hg(II), the thermodynamic parameters were also computed (ΔG°, ΔH°, and ΔS°).

The ability of MA@NBAL to extract Hg(II) ions in presence of different ionic strengths (10–100 mg NaCl) was determined by mixing with the nanobiosorbent (1.0 g/L). Then, 10.0 mL Hg(II) solutions (5 and 10 mmol/L at pH 6.0) were added to NaCl under the same experimental steps listed above, and Equation (1) was used to compute the capacity value (mg/g) values.

The potential applications of MA@NBAL nanobiosorbent in Hg(II) ion removal from various contaminated real water matrices, such as wastewater, sea water, and tap water, were determined under the batch condition. Various water samples (10.0 mL each) were spiked with the desired Hg(II) concentrations (2.0 and 5.0 µg/mL), mixed with 2.0 g/L of MA@NBAL and shaken for 30.0 min period. Finally, the unadsorbed Hg(II) ions were separated, as previously described. These steps were conducted in triplicate to determine the removal percentage (%*R*) of Hg(II) according to Equation (2).

(2) $$\%R=\frac{\left(C_{o}-C_{t}\right)}{C_{t}}\times 100$$

## 3. Results and Discussion

### 3.1. Structural and Surface Characterization

**Figure 1:**
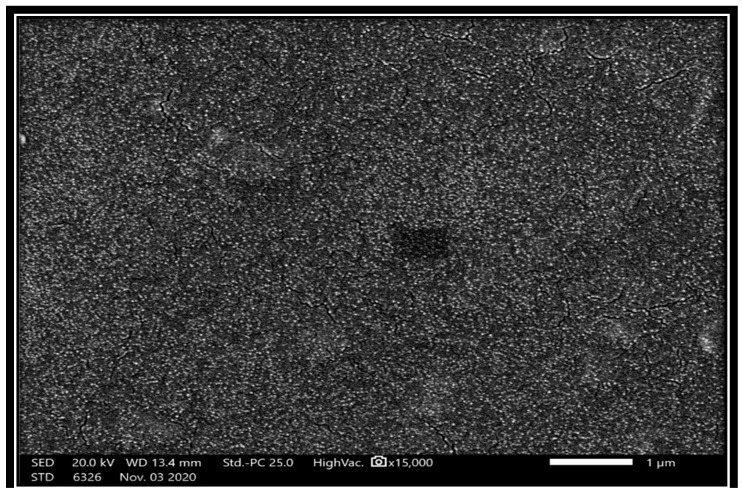

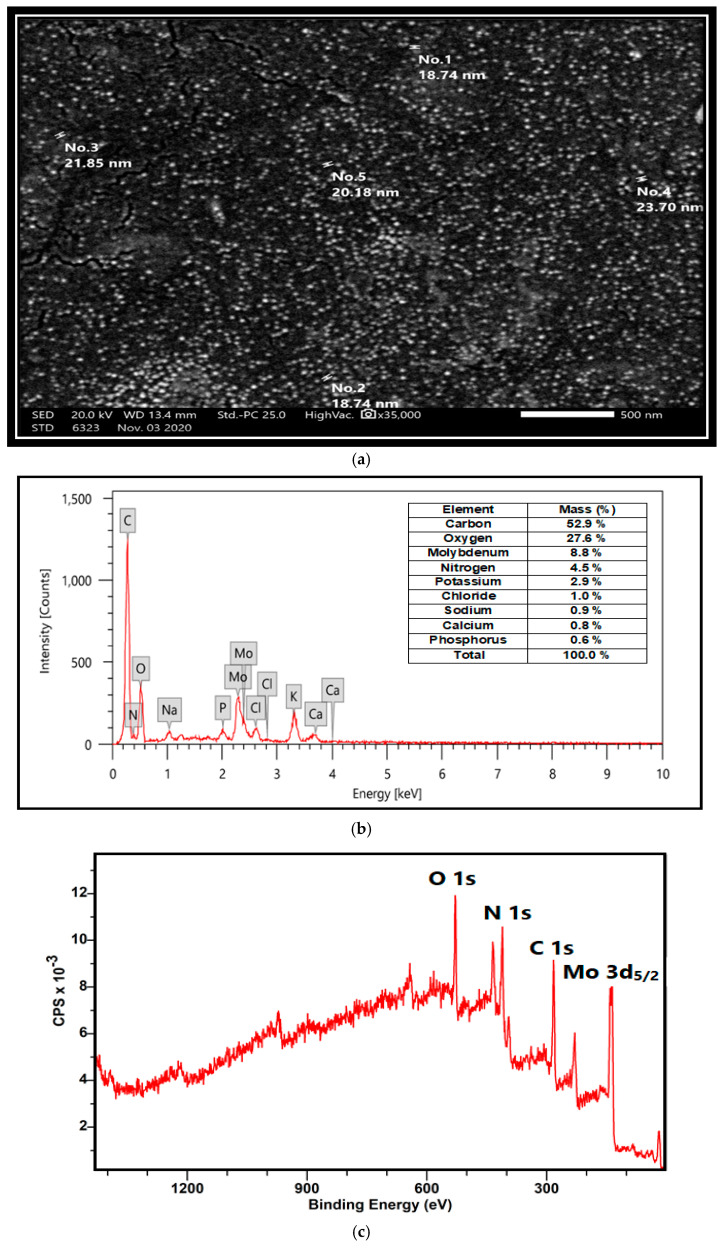

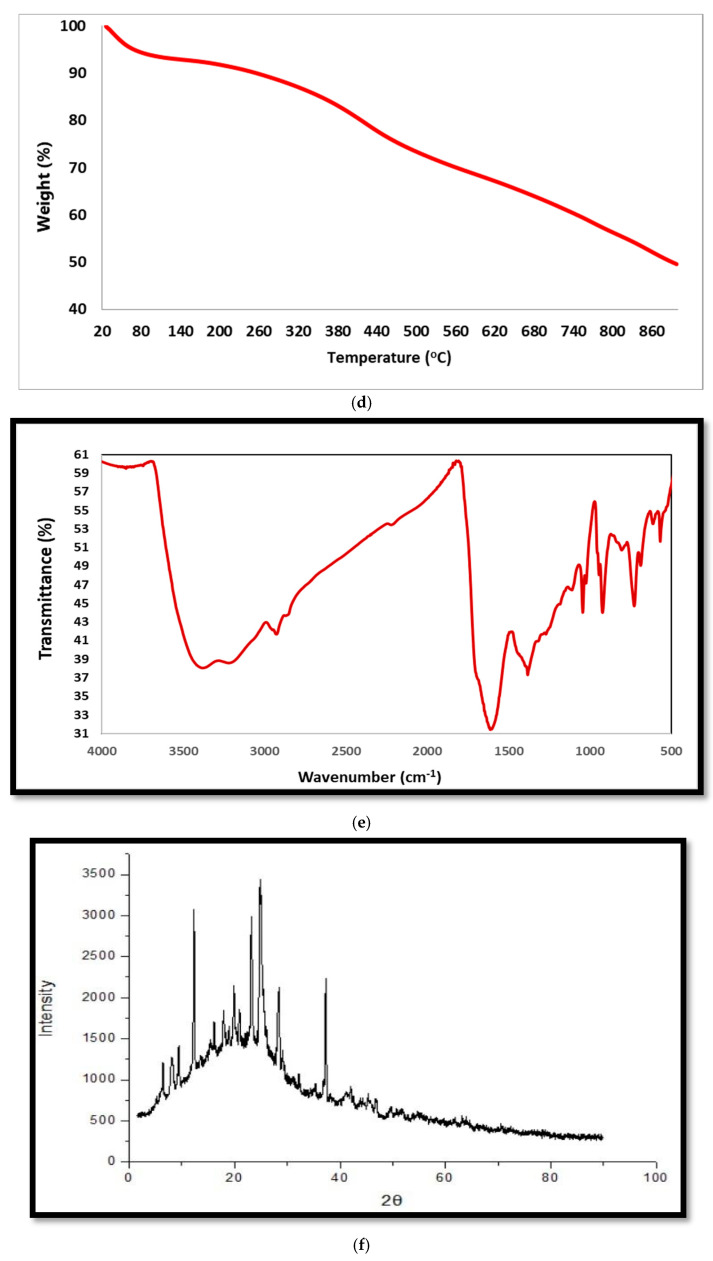

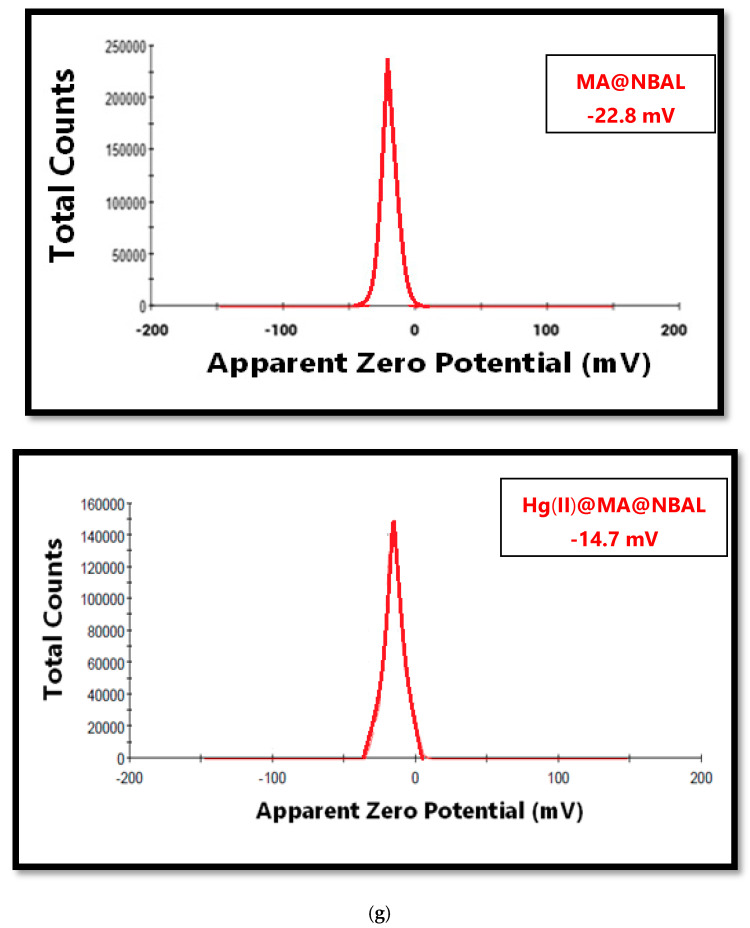
(**a**) SEM images and particle size of MA@NBAL nanobiosorbent. (**b**) EDX analysis and elemental composition of MA@NBAL nanobiosorbent. (**c**) XPS survey of MA@NBAL nanobiosorbent. (**d**) TGA and thermal decomposition of MA@NBAL nanobiosorbent. (**e**) FT-IR spectrum of MA@NBAL nanobiosorbent. (**f**) XRD pattern of MA@NBAL nanobiosorbent. (**g**) Zeta potential measurements of MA@NBAL and Hg(II)@MA@NBAL.

A SEM image of the MA@NBAL nanobiosorbent with its particle size range is illustrated in Figure 1a. A homogenous and good spherical particle distribution is evident in the confirmed nanostructure of produced particles at 18.74 nm to 23.70 nm, as represented in Figure 1. Moreover, Figure S1 (Supplementary Materials) shows the TEM image of the MA@NBAL nanobiosorbent. The EDX analysis and percentage of the elemental composition of MA@NBAL are outlined in Figure 1b, which refer to the major and minor elements in the assembled nanobiosorbent. It is clear from the percentage composition of various elements that carbon (52.9%), oxygen (27.6%), molybdenum (8.8%), and nitrogen (4.5%) are the four highest elements existing in MA@NBAL, providing a total (%) corresponding to 93.8%, as these elements are mainly related to both constituting units in MA@NBAL. In addition, several other minor elements were found to exist in the assembled nanobiosorbent, such as potassium, chloride, sodium, calcium, and phosphorus, comprising 2.9%, 1.0%, 0.9%, 0.8%, and 0.6%, respectively, which are mainly generated from the biochar material. For comparison, the EDX of pristine NBAL was also acquired to confirm the major constituting elements related to C (78.8%) and O (17.0%), as well as other minor elements such as Na, K, Mg, Ca, Cl, and P (total of 4.4%).

The XPS analysis was acquired for MA@NBAL to confirm the various elements incorporated into the assembled nanobiosorbent, as depicted in Figure 1c. This refers to several sharp peaks related to molybdenum, oxygen, and carbon constituents. The centered peak at a binding energy of 279–286 eV refers to the carbon functional groups in MA@NBAL nanobiosorbent C 1s, which is correlated to the incorporated C1s-π* transition of COH, COOH, C=O, C-O moieties, phenolic C, and substituted aromatic carbon. Meanwhile, the C1s-3p-σ* peak is due to aliphatic C-H in the forms of CH_3_, CH_2_, and CH bonds to affirm the presence of such carbonaceous moieties. On the other hand, the peak centered at a binding energy of 524–532 eV corresponding to O 1s is due to the presence of some oxygenated functionalities, including C-O, C=O, COO, and O-H [40]. Moreover, the data collected from the XPS of MA@NBAL nanobiosorbent also confirm the existence of a Mo(VI) peak with a binding energy (231–232 eV) for Mo 3d_5/2_, as shown in Figure 1c, which affirms that Mo(VI) did not experience a redox reaction and thus kept its original valence structure in the assembled MA@NBAL nanobiosorbent [41]. The nitrogen peak detected at the range of 398–402 eV is due to N 1s, which is attributed to nitrogen atoms in the C–N, C=N, N-N, and N-O functionalities [42].

The TGA and thermal decomposition of the MA@NBAL nanobiosorbent at the range of temperature from 25 to 900 °C are illustrated in Figure 1d. Three different degradation stages are characterized in the entire temperature range. These are characterized at (i) 25–145 °C, (ii) 145–460 °C, and (iii) 460–900 °C, with weight percentage losses corresponding to 6.8%, 17.7%, and 23.4%, respectively. Step (i) mainly refers to the volatilization and surface evaporation of adsorbed water molecules, while step (ii) denotes the initial and partial decomposition of the NBAL biochar moiety with the release of carbonaceous, oxygenated, and nitrogenated functionalities. Step (iii) refers to the final thermal degradation process of NBAL biochar [43]. The remaining weight percentage of MA@NBAL nanobiosorbent (47.9%) after thermal heating to 900 °C is basically related to the surface-loaded molybdic acid (MA), along with other stable metal functionalities in the NBAL biochar.

FT-IR analysis is generally used to characterize the surface functional groups in newly assembled materials, such as MA@NBAL. Therefore, Figure 1e illustrates and provides more details about incorporated functionalities by doping MA into NBAL. Several related characteristic functional groups to the NBAL are evident based on the assigned peaks at 3210–3420 cm^−1^ for O-H groups, 2933 and 2959 cm^−1^ for the C–H group, 1627 cm^−1^ for the C=O group, 1593 cm^−1^ for the C=C group, and 1115–1200 cm^−1^ for the pyrone group [44]. In addition, Figure 1e also refers to the presence of other ascribed functional groups to the molybdic acid at 990 cm^−1^ (Mo=O), 815 cm^−1^ (Mo–O–Mo), and 660 cm^−1^ (Mo_2_O_2_) [45]. Therefore, the existence of these functionalities provides good evidence of efficient and successful assembly of the target MA@NBAL nanobiosorbent.

The lattice structure of MA@NBAL was also investigated via the XRD technique, as depicted in Figure 1f. The nanobiosorbent was characterized by the appearance of various related diffraction peaks of MA at 2θ 12.4°, 14.8°, 18.5°, 22.1°, 24.9°, 28.1°, and 38.3° [36]. Moreover, the appearance of some overlapped, broad low peaks at the 2θ range between 16.6° and 37.5° is basically attributed to the amorphous character of NBAL biochar [44]. Hence, the existence of these XRD peaks in the assembled MA@NBAL confirms the successful preparation of this nanobiosorbent.

The surface characteristics of MA@NBAL nanobiosorbent were determined by using the BET method to identify the values related to the surface area, total pore volume, and mean pore diameter—15.38 m^2^/g, 3.78 cm^3^/g, and 64.0 nm, respectively. It is important to point out that the low surface area is not a reflection of the capability of MA@NBAL nanobiosorbent to react and bind with cationic pollutants such as Hg(II) ions. This assumption is mainly based on the presence of several surface-loaded and reactive functional groups, such as carboxyl, hydroxyl, and other nitrogenous moieties, which favor the cationic capture of metal ions via cation exchange and/or complex formation mechanisms [10], and this will be explored and disused in following sections.

To verify the effective surface charge on MA@NBAL nanobiosorbent, the zeta potential was determined, as represented in Figure 1g. The identified zeta potential of MA@NBAL nanobiosorbent was detected at −22.8 mV, indicating the incorporated surface negative charge via loading of molybdic to establish a strong affinity for binding with the target positively charged Hg(II) pollutant. Additionally, the adsorbed Hg(II) ions on the surface of MA@NBAL to produce Hg(II)@MA@NBAL were also analyzed using the zeta potential technique to figure out and determine the final surface charge of the produced material, as represented in Figure 1g. This was identified to exhibit a direct shift in zeta potential to a less negative value at −14.7 mV due to the efficient binding of positively charged Hg(II) ions.

### 3.2. Adsorption of Hg(II) by MA@NBAL Nanobiosorbent Under Diverse Optimized Parameters

#### 3.2.1. Reaction pH

The pH’s influence on the adsorption process of Hg(II) ions onto MA@NBAL nanobiosorbent was optimized in this study owing to its direct impact as a main factor on the Hg(II) ion-removal process. Generally, the pH of the interaction solution is known and expected to affect the MA@NBC surface characteristics, including charge, active sites, solubility, and others, as well as the significant influence on existing Hg(II) ions and its ionization, hydrolysis, and complex formation with the functional groups on MA@NBAL [46]. To investigate this factor, different pH solutions (2.0–6.0) were applied to figure out and conclude the capacity of removed Hg(II) by this nanobiosorbent. The batch experimental study was followed by using two Hg(II) concentrations (5.0 and 10.0 mmol/L). Table 1 refers to the collected results from the pH factor by providing the characterized mg/g Hg(II) values via direct reaction with MA@NBAL nanobiosorbent. It was confirmed that the Hg(II) ion removal tendency was enhanced by increasing pH to provide the maximum capacity efficiency at pH 6.0. A gradual enhancement in the capacity value was also detected by increasing the pH from 2.0 to 6.0. For 5.0 mmol/L, the adsorption capacity was 140.4 mg/g, while in the case of 10.0 mmol/L, it corresponded to 1444.25 mg/L. This trend can be directly related and interpreted on the basis of the loaded surface charge on MA@NBAL, as well as the present Hg(II) ions in the aqueous solution, which are mainly dependent on the solution pH. The surface of MA@NBAL is loaded with numerous hydroxyl (–OH) and carboxyl (–COOH) active functional groups, and at low pH levels (pH 2.0–4,0), the surface becomes protonated to acquire positively charged ions (–OH_2_^+^ and –COOH_2_^+^) to exhibit a low tendency to bind with Hg(II) cations [47]. On the other hand, when increasing the solution pH from > 4.0 to 6.0, the concentration of H^+^ ions declined, and the surface functional groups on MA@NBAL thus became more difficult to protonate, providing higher Hg(II) removal efficiency. Finally, the results collected from the pH dependence denote that pH 6.0 could be concluded as the optimum condition for Hg(II) removal from aqueous solutions.

#### 3.2.2. Reaction Time and Kinetics Evaluation

**Figure 2:**
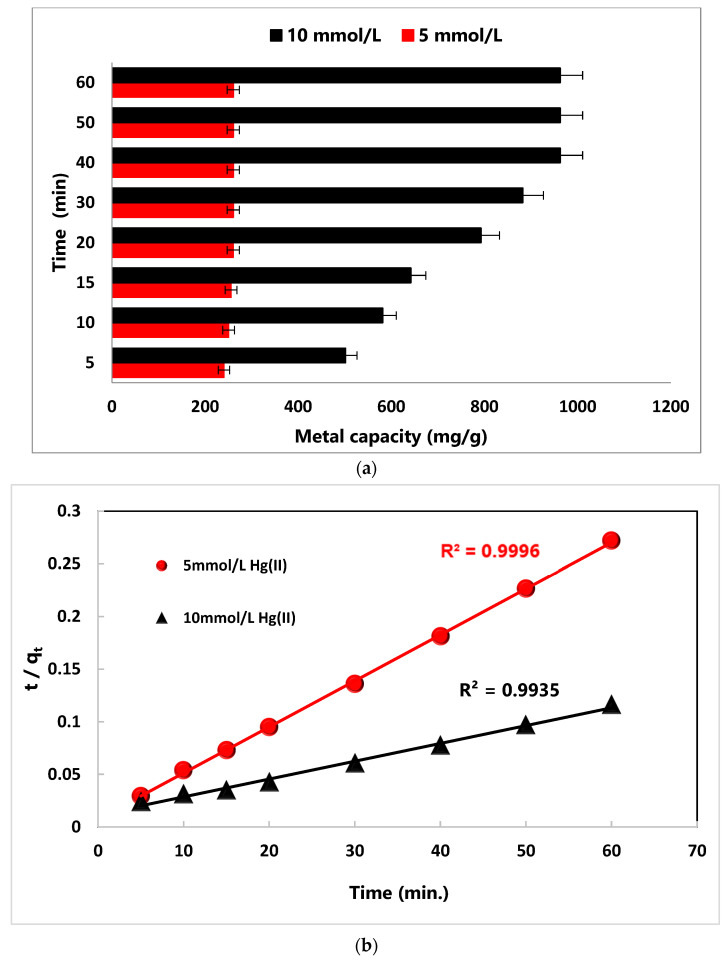
(**a**) Effect of reaction time (min) on Hg(II)-removal capacity (mg/g) using MA@NBAL nanobiosorbent. (**b**) Pseudo-second order model for Hg(II) removal using MA@NBAL nanobiosorbent.

Figure 2a refers to the capacity of Hg(II) characterized by the investigated MA@NBAL nanobiosorbent at different reaction times under various initial concentrations of Hg(II) (5.0 and 10.0 mmol/L). The adsorption of Hg(II) was identified to follow a general fast rate at the beginning owing to the high number of available vacant and free reactive surface sites on MA@NBAL for binding with Hg(II) ions [48]. As these sites became more occupied over time, the uptake rate gradually declined down until an equilibrium plateau was established. It is evident for 5.0 mmol/L concentration of Hg(II) that a gradual increase in the capacity from 240.7 mg/g to 255.8 mg/g was developed by the contact time raising from 5 min to 15 min. After that, the Hg(II) capacity determined using MA@NBAL was found to stabilize at a reaction time of ≥20 min and reach the equilibrium condition at 260.8 mg/g. Similarly, the adsorption of the 10.0 mmol/L concentration of Hg(II) onto MA@NBAL was found to gradually increase from 501.7 mg/g to 882.6 mg/g by increasing from 5 min to 30 min. This is because equilibrium conditions took up to 40 min to be established, leading to a capacity value of 962.8 mg/g. The high mg/g capacity values at the equilibrium conditions might result from the complete binding of Hg(II) with MA@NBAL to confirm that all reactive groups on the nanobiosorbent were completely combined with Hg(II) ions [49,50].

A kinetics evaluation of the collected practical data to characterize the possible mechanism types, as well as the mass transport and chemical reaction, are generally applied on the basis of the contact time factor to determine the best fitting conditions for binding and uptake of Hg(II) [51,52]. To investigate and find out the best fitting mechanism(s) in this work, the following kinetic models (*p*seudo-first-order, the *p*seudo-second-order, Elovich, and intra-particle diffusion) were applied to the collected removal data of Hg(II) onto MA@NBAL under the conditions of initial Hg(II) concentrations (5 and 10 mmol/L) and pH 6.0 at 25.0 °C: The equations and parameters identified by these models are outlined in Table S2 (Supplementary Materials), while the graphical illustrations are shown in Figure 2b and Figure S2 (Supplementary Materials). The predicted R^2^ and q_e_ values are the two main parameters used to validate the best-fitting kinetics (Table 2). The R^2^ computed by the *p*seudo-first-order corresponded to 0.973 and 0.981 using 5.0 and 10.0 mmol/L Hg(II)), respectively. These two R^2^ are lower than the ones computed by the *pseudo*-first-order (R^2^ = 0.999 for 5.0 mmol/L Hg(II) and 0.993 for 10.0 mmol/L Hg(II)), confirming high fitting to the experiment. Moreover, the computed q_e(cal)_ = 227.27 mg/g (q_e(exp)_ = 220.64 mg/g) by 5.0 mmol/L Hg(II) and q_e(cal)_ = 528.23 mg/g (q_e(exp)_ = 516.52 mg/g) by 10.0 mmol/L Hg(II) point out the additional best fitting by this model. The intra-particle diffusion expression, referred to as R^2^ = 0.788 for 5.0 mmol/L Hg(II) and 0.776 for 10.0 mmol/L Hg(II), confirms that it was poorly fitted to the adsorption results. Moreover, the R^2^ values concluded using the Elovich model generally refer to heterogeneous surfaces, and these were found to be 0.902 and 0.898 by using 5.0 mmol/L and 10.0 mmol/L Hg(II), respectively, also suggesting less fitting than the *pseudo*-second-order. However, the adsorption rate values (α) listed by the Elovich model refer to the rapid and effective adsorption process of Hg(II) via MA@NBAL [53]. Therefore, the concluded values of the *pseudo*-second-order point out the best match for Hg(II) ion adsorption onto MA@NBAL, confirming that this process is related to chemisorption [54]. Therefore, the interactions of Hg(II) with MA@NBAL were mainly accomplished using cation-exchanger and complex-formation mechanisms.

#### 3.2.3. MA@NBAL Nanobiosorbent Dosage

**Figure 3:**
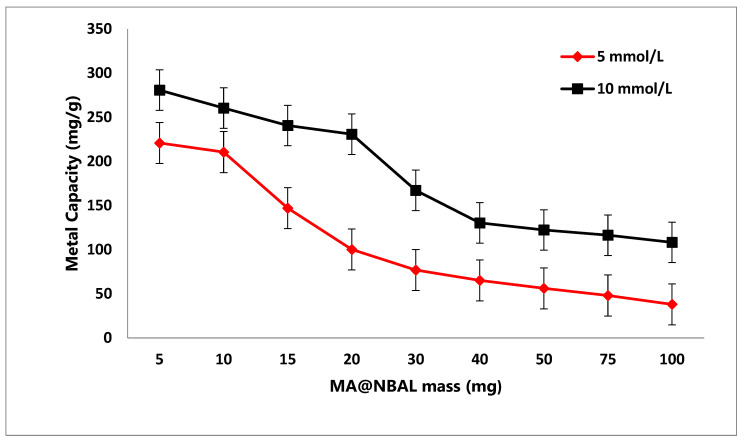
MA@NBAL nanobiosorbent dosage (mg) on Hg(II) removal capacity (mg/g).

The removal of various pollutants from water is heavily dependent on the adsorbent dosage as a significant affecting parameter. Moreover, the consumption of small quantities of the investigated adsorbent is profitable from an economical point of view [55]. The adequate optimum quantity of MA@NBAL nanobiosorbent for the adsorption of Hg(II), 5.0 and 10.0 mmol/L of Hg(II), was evaluated by using the selected MA@NBAL dosages in the range of 5.0–100.0 mg. The results of these experiments are depicted in Figure 3, which denotes that the Hg(II) capacity reached the highest values at 220.9 and 280.8 mg/g for 5.0 and 10.0 mmol/L Hg(II) by using 5.0 mg of MA@NBAL as the lowest investigated dosage of MA@NBAL nanobiosorbent. This behavior is mainly correlated to the increase in the metal ion ratio versus the mass of the adsorbent. It is also evident that the characterized minimum mg/g values of Hg(II) were identified as 38.1 and 108.3 by using 100.0 mg of the MA@NBAL nanobiosorbent for 5.0 and 10.0 mmol/L Hg(II) concentrations, respectively, and this trend is directly devoted to the decrease in the metal ion ratio versus the mass of the MA@NBAL nanobiosorbent [56].

#### 3.2.4. Initial Concentration of Hg(II) and Adsorption Isotherms

**Figure 4:**
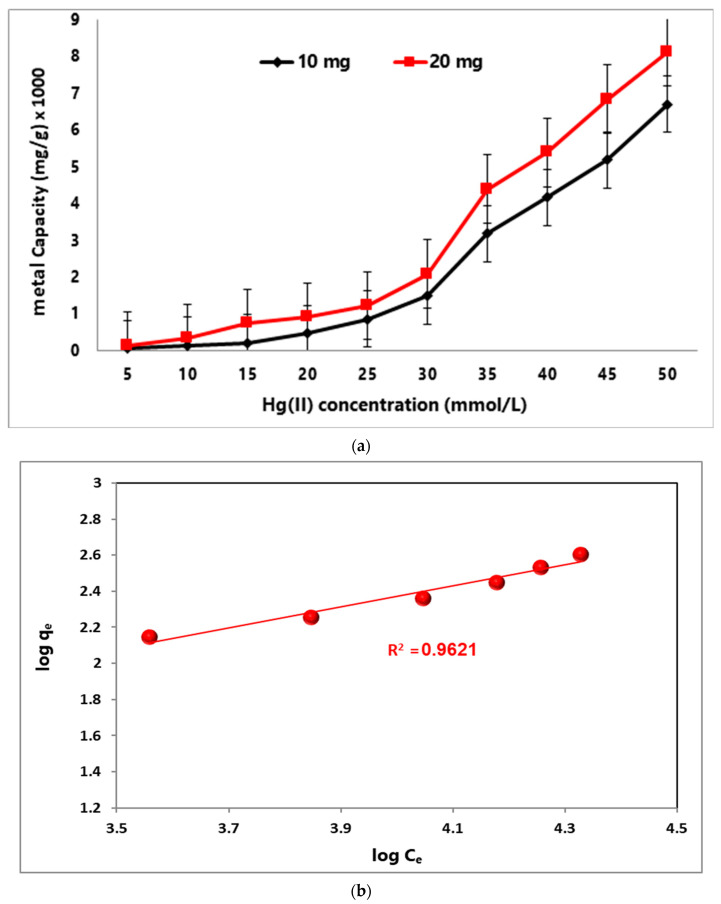
(**a**) Effect of initial Hg(II) concentration on removal capacity (mg/g) of MA@NBAL nanobiosorbent. (**b**) Freundlich adsorption isotherm model for Hg(II) removal via MA@NBAL nanobiosorbent.

The initial Hg(II) concentration is a valuable factor in determining the capacity of the MA@NBAL nanobiosorbent. This factor was investigated by using 5–50 mmol/L Hg(II) solutions at pH 6 in the presence of 10 and 20 mg of MA@NBAL nanobiosorbent, and the data collected from this study are represented in (Figure 4a). It is evident that the highest adsorption capacity values were characterized by using 50 mmol/L of Hg(II) ions, 8145 mg/g and 6719 mg/g, with 10 and 20 mg of MA@NBAL nanobiosorbent, respectively. The above behavior is basically attributed to the high Hg(II) ratio versus surface functionalities on MA@NBAL nanobiosorbent [57]. Moreover, the lowest adsorption capacity values were found by using 5 mmol/L of Hg(II) ions, 140 mg/g and 65 mg/g, with 10 mg and 20 mg of MA@NBAL nanobiosorbent, respectively, due to the low ratio of metal ions versus surface-accessible functional groups.

To explain the interaction between MA@NBAL and Hg(II) pollutants, adsorption isotherms are generally implemented to figure out the possible equilibrium condition for Hg(II) ion reaction in solution (C_e_) versus the amount already adsorbed by the MA@NBAL nanobiosorbent (q_e_). In general, the adsorption equilibrium is reached when the Hg(II) ions adsorbed onto the nanobiosorbent are in balance with the remaining species in the solution [58,59]. Therefore, the equilibrium data for removing Hg(II) onto MA@NBAL were discussed using these expressions (Langmuir, Freundlich, Temkin, and Dubinin–Radushkevich) with initial Hg(II) metal ion concentrations (5.0–50.0 mmol/L) at 25 °C and pH 6.0. Table S3 (Supplementary Materials) lists the parameters concluded for the sorption process. It is evident that the Freundlich model (Figure 4b) represents the sorption process more effectively, with the highest *R*^2^ = 0.962, using 20 mg of MA@NBAL nanobiosorbent to verify the surface coverage of MA@NBAL with Hg(II) ions in a heterogeneous surface with unequal free active binding sites [60]. The adsorption of Hg(II) ions onto MA@NBAL nanobiosorbent was confirmed to follow the Freundlich according to the postulates of this model via multilayer formation. However, the intensity constant was found (n > 1) to refer to favorable adsorption by the MA@NBAL nanobiosorbent [61]. Moreover, the characterized K_F_ (1.119 L/mg) also refers to a high capacity of Hg(II) onto MA@NBAL. The identified maximum capacity (q_max_) of Hg(II) onto MA@NBAL using the Langmuir expression was found to be 666.6 mg/g (Table 3 and Figure S3, Supplementary Materials), which indicates a good binding interaction between Hg(II) ions and MA@NBAL at pH 6.0. The dimensionless separation factor (*R_L_*) was computed using Equation (3) [62].

(3) $$R_{L}=\frac{1}{1+K_{L}C_{o}}$$

where *C_o_* (mg/L) denotes the Hg(II) initial concentration, and *K_L_* (L/mg) is the Langmuir constant, while *R_L_* shows the feasibility of the adsorption process. If *R_L_* is greater than zero but less than 1, this indicates a favorable process. If R_L_ is greater than 1, it is unfavorable. If *R_L_* = 0, it is irreversible, and finally, if *R_L_* = 1, it indicates a linear process [63]. The listed values of *R_L_* in Table 3 are in the range of 0.936–0.988, confirming the favorable binding of Hg(II) onto MA@NBAL. The Temkin isotherm [64] postulated a decrease in emitted heat during the adsorption process, providing a linear behavior due to more Hg(II) ions adsorbed by MA@NBAL. This model can also be used to predict if this reaction is controlled via physical or chemical process, and the Temkin computed parameters are outlined in Table 3. However, Equation (4) is employed to conclude *b_T_* in kJ/mol as the Temkin equilibrium constant for interpretation of the adsorption heat and/or the adsorption bonding energy.

(4) $$b_{T}=\frac{RT}{B}$$

where *B* = Temkin constant, *R* = 8.314 × 10^−3^ kJ mol^−1^ K^−1^, and *T* = temperature (K). Therefore, a chemisorption process is favored. The identified R^2^ = 0.886 reveals that the Hg(II) adsorption onto MA@NBAL nanobiosorbent was fitted less to the Temkin model than the Freundlich expressions. Moreover, the D-R isotherm evaluated the equilibrium of Hg(II), and the sorption energy (Es in kJ/mol) was calculated using equation Equation (5), which is a parameter that is very helpful in predicting the type of adsorption mechanism.

(5) $$E_{s}=\frac{1}{\sqrt{2K_{ad}}}$$

when *E_s_* < 8 kJ/mol, a physical adsorption takes place, while *E_s_* = 8–16 kJ/mol points to chemisorption [65]. The characterized *E_s_* value (0.521 kJ/mol) refers to physisorption. However, the D-R isotherm expression provided less of a match based on the detected R^2^ = 0.688.

Finally, the Freundlich model (Figure 4b) should be listed as the most suitable expression to represent the chemisorption of Hg(II) ions onto a MA@NBAL nanobiosorbent, and it is more effective in verifying heterogeneous surface coverage with unequal free active binding sites. Morefore, the detected high adsorption capacity (1444.25 mg/g) is certainly produced by the successive adsorption of Hg(II) ions on the surface of MA@NBAL to favor the formation of a multilayer. This conclusion was also confirmed by investigating the non-linear adsorption expression, as represented in Figure S4 (Supplementary Materials).

#### 3.2.5. Reaction Temperature and Thermodynamic Parameters

**Figure 5:**
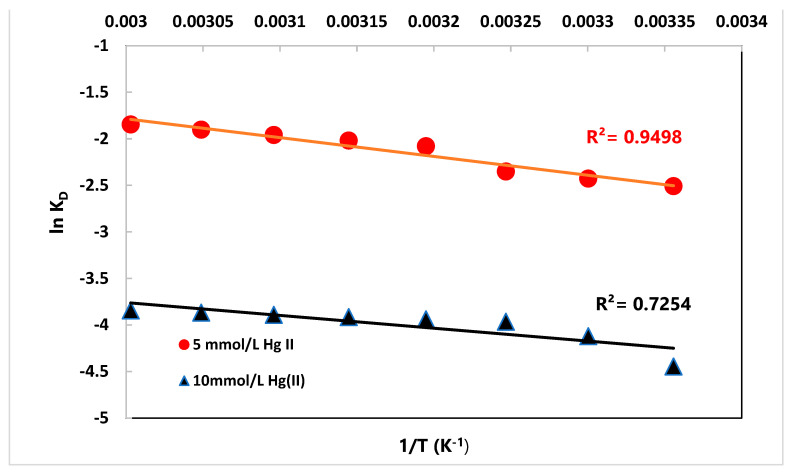
CorrectedVan’t Hoff’s plot for removal of Hg(II) using MA@NBAL nanobiosorbent.

The temperature’s impact on removing various pollutants was characterized and reported to exhibit a significant role and influence on the removal percentage and capacity values of the target pollutant [66]. This study was generally performed to obtain a good idea of the sorption process as either exothermic or an endothermic. The adsorptive removal percentage of Hg(II) onto MA@NBAL was tested at 293–333 K by using 5.0 and 10.0 mmol/L Hg(II), and the collected results are outlined in Table 4. The adsorptive removal percentages of Hg(II) onto MA@NBAL were enhanced by a temperature increase, which suggests an endothermic mechanism. The maximum Hg(II) capacities were characterized as 110.3 and 481.4 mg/g by using 5.0 and 10.0 mmol/L of Hg(II), respectively, at 333 K. Thus, the developed MA@NBC was able to remove Hg(II) in a wide range of reaction temperatures. To determine whether the adsorption of Hg(II) by MA@NBAL was based on a spontaneous or nonspontaneous process, it was important to compute the thermodynamic parameters of this process in the forms of ΔG°, ΔH°, and ΔS° according to Equations (6)–(8) below [67].

(6) $$\Delta G^{o}=-RT\ln K_{d}$$

(7) $$\Delta G^{o}=\Delta H^{o}-T\Delta S^{o}$$

(8) $$\ln K_{d}=\frac{\Delta S^{o}}{R}-\frac{\Delta H^{o}}{RT}$$

where *T* = (K) and *K_d_* (L/g) = equilibrium constant calculated from Equation (9).

(9) $$K_{d}=\frac{q_{e}}{C_{e}}$$

where *q_e_* (mg/g) refers to the Hg(II) equilibrium capacity of MA@NBAL, and *C_e_* (mg/L) is the Hg(II) equilibrium concentration. The values of ΔH° and ΔS° can be calculated from Van’t Hoff’s plot (Figure 5), as listed in Table 5. The ΔG° values decreased from 6.214 to 5.110 J/mol and from 11.014 to 10.639 kJ/mol with 5.0 and 10.0 mmol/L of Hg(II), respectively, by enhancing the temperature from 293 to 333 K. The positive ∆G^o^ values for the reaction between Hg(II) ions and MA@NBAL refer to the requirement of an input of energy in order to take place via a nonspontaneous endothermic process [68]. Moreover, the positive signs of ΔH° (16.795 (5 mmol/L) and (11.466 kJ/mol 10.0 mmol/L) indicate the endothermic behavior for Hg(II) ions onto MA@NBAL. Moreover, the characterized positive ΔS° values were 35.392 and 31.149 J/mol K with 5.0 and 10.0 mmol/L Hg(II) concentrations, respectively, indicating an increased randomness, during adsorption, at the interface between MA@NBAL and Hg(II) ions in the aquatic condition [69].

#### 3.2.6. Ionic Strength and Regeneration of MA@NBAL After Initial Removal of Hg(II) Pollutants

**Figure 6:**
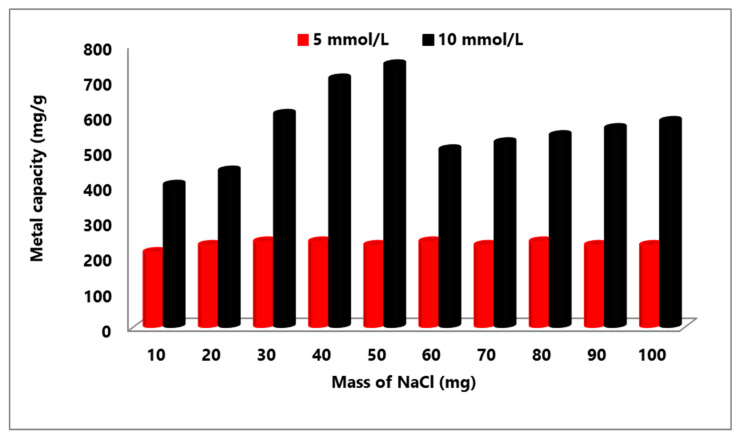
Effect of ionic strength on Hg(II)-removal capacity (mg/g) via MA@NBAL nanobiosorbent.

The ability of a sorbent to collect metal ions from solutions may be dependent on the presence of high or low ionic strength of other competing ions. Therefore, the values of the adsorptive removal capacity of Hg(II) by MA@NBAL were examined and determined in the presence of ionic strength values (10–100 mg of NaCl), and this was tested by mixing the nanobiosorbent (10.0 mg) with 10.0 mL Hg(II) solutions (5 and 10 mmol/L at pH 6.0; the results are plotted in Figure 6). As illustrated, the characterized metal capacity values in the case of 5 mmol/L Hg(II) were slightly changed to arrange from 210.6 mg/g to 240.7 mg, which are very close to the estimated capacity value of Hg(II) in the absence of NaCl (260.8 mg/g). The impact of the ionic strength via NaCl on the removal efficiency of MA@NBAL for 10 mmol/L of Hg(II) was found to contribute more to lowering the capacity values to 401.1 and 441.3 mg/g in the presence of 10 and 20 mg of NaCl, respectively. However, in the presence of 30–50 mg of NaCl, the capacity values of Hg(II) were identified to enhance, providing the range 601.8–742.2 mg/g. Generally, the ionic charge and nature of surface functionality groups on the nanobiosorbent are the two main factors for increasing or decreasing the efficiency of target metal ions such as Hg(II) and interfering with Na(I) ions on MA@NBAL [70]. For example, the interfering sodium ions are expected to load the surface of MA@NBAL nanobiosorbent with positive charges that inhibit and retard Hg(II) binding, as in the case of 10–20 mg of NaCl. However, Hg(II) ions may favor reacting with the surface of the MA@NBAL nanobiosorbent via a cation-exchange mechanism upon complete saturation of the surface with Na(I) ions at 30–50 mg of NaCl.

The regeneration of MA@NBAL after the initial removal of Hg(II) ions was also completed in this work to figure out its application validity in the successive removal of Hg(II) pollutants from wastewater. The Hg(II) initially loaded onto MA@NBAL was subjected to treatment and desorption from the surface by using 0.01 mol/L HCl. The recycled MA@NBAL was then dried and reapplied to remove Hg(II) from the aquatic sample. The detected percentage removal of Hg(II) ions was found to correspond to 93.14% to provide excellent evidence for the possibility of excellent stability and reusability of the investigated MA@NBAL nanobiosorbent.

#### 3.2.7. Potential Applications of MA@NBAL in Recovery of Hg(II) from Real Water Matrices

Water is well-documented and reported to represent a big issue in the future in terms of treatment and purification [71]. Therefore, this work aimed to investigate the applicability of MA@NBAL in the adsorptive recovery of Hg(II) ions from some contaminated real water matrices, which represents an essential final procedure to validate the presented method. Three different water samples were used in this investigation, with the specifications listed in Table S4 (Supplementary Materials). The removal of Hg(II) was accomplished in contaminated wastewater (El-max Bay), sea water, and tap water, with 2 and 5 µg/mL Hg(II), adjusted to the optimum pH 6.0 condition. The data collected from this investigation indicate removal efficiencies of Hg(II) from tap water of 93.5–98.1%; from wastewater, 92.4–95.5%; and from sea water, 91.2–97.7%, in the diverse contaminated samples. Therefore, the listed results confirm and refer to the acceptable applicability of the MA@NBAL nanobiosorbent for the effective adsorptive removal of Hg(II) pollutants from real aquatic sources with high performance and efficiency.

## 4. Conclusions

The target MA@NBAL nanobiosorbent was developed via a facile chemical-microwave procedure to verify its potential effectiveness in successfully removing Hg(II) pollutants from diverse contaminated water matrices. The SEM revealed homogenous and spherical nanostructural particles 18.74 nm to 23.70 nm; the EDX data referred to major elemental compositions, including carbon (52.9%), oxygen (27.6%), molybdenum (8.8%), and nitrogen (4.5%); and the XPS investigation approved the existence of these four elements. The sorption process and related experimental control parameters were tested to verify the optimum uptake of Hg(II) ions onto MA@NBAL nanobiosorbent by using the batch mode. The maximum values of Hg(II) removal by using a 10.0 mmol/L concentration of Hg(II) corresponded to 1444.25 mg/g at pH 6.0, an equilibrium time of 30.0 min, and a nanobiosorbent dosage of 20 mg. The contribution of ionic strength by using 30–50 mg of NaCl was found to influence the capacity values of Hg(II): 601.8–742.2 mg/g. The undertaken kinetic studies confirmed the best fitting to the *pseudo*-second model, suggesting the possible chemical binding interaction via cation exchange and complex formation mechanisms. Evaluation of the isotherm models indicated that the Langmuir and Freundlich models were validated to exhibit the two most fitting models. The computed thermodynamic parameters for Hg(II) removal on MA@NBAL nanobiosorbent referred to thermodynamically endothermic and feasible behavior with the spontaneous adsorption of Hg(II) via a high preference. The investigated MA@NBAL was confirmed as a promising nanobiosorbent for Hg(II) uptake from different contaminated samples, providing removal efficiencies of Hg(II) of 93.20–97.9% from tap water, 92.1–95.4% from wastewater and 94.8–97.7% from sea water. MA@NBAL exhibited excellent reusability and stability based on the detected 93.14% removal of Hg(II) via a recycled nanobiosorbent. Finally, the developed MA@NBAL was found to be highly promising by displaying excellent Hg(II) ion adsorptive removal efficiency versus other adsorbents, as compared and listed in Table 6 [71,72,73,74,75,76,77,78].

## Figures and Tables

**Table 1:** Effect of pH factor (conditions: pH 2–6, 5 and 10 mmol/L^−1^ Hg(II) concentration, time of 30 min, Temp = 25 °C, and mass of MA@NBAL = 20 mg).

Initial Hg(II) Concentration	Determined Hg(II) Capacity Values (mg/g) by MA@NBAL at Different pHs				
	pH 2	pH 3	pH 4	pH 5	pH 6
5 mmol/L	110.3	90.3	80.2	140.4	140.4
10 mmol/L	481.4	842.5	320.9	1303.8	1444.3

**Table 2:** Kinetic parameters computed for adsorption of Hg(II) onto MA@NBAL by different models.

Kinetic Model	Hg(II)	
	5 mmol/L	10 mmol/L
*Pseudo*-first-order		
q_e_ (mg g^−1^)(exp.)	220.64	516.52
q_e_ (mg g^−1^)(calc.)	93.822	523.11
k_1_ (min.^−1^)	0.1135	0.1131
R^2^	0.973	0.981
*Pseudo*-second-order		
q_e_ (mg g^−1^)(exp.)	220.64	516.52
q_e_ (mg g^−1^)(calc.)	227.27	588.23
k_2_ (g mg^−1^ min.^−1^) × 10^2^	2.58 × 10^−3^	0.247 × 10^−3^
R^2^	0.999	0.993
Intraparticle diffusion		
K_id_ (mg·g^−1^ min^−1/2^)	8.738	51.013
C	162.77	177.65
R^2^	0.788	0.776
Elovich		
α (mg g^−1^ min^−1^)	16,425.78	186.366
β (mg g^−1^)	0.047	0.008
R^2^	0.902	0.898

**Table 3:** Adsorption isotherm parameters computed by different models.

Isotherm Model	Isotherm Parameters	Computed Values
Langmuir	q_max_ (mg/g)	666.6
	b (L mg^−1^)	5.675 × 10^−5^
	R_L_	0.936–0.988
	R^2^	0.810
Freundlich	n	1.724
	K_f_ (L·mg^−1^)	1.119
	R^2^	0.962
Temkin	a_T_ (L·g^−1^)	6.094 × 10^−4^
	b_T_ (J/mol)	17.826
	B	138.98
	R^2^	0.886
Dubinin–Radushkevich	q_s_ (mg/g)	305.82
	K_ad_ (mol^2^/j^2^)	1.839
	E_s_ (kJ mol^−1^)	0.521
	R^2^	0.688

**Table 4:** Effect of reaction temperature on Hg(II) removal (conditions: pH 6, time of 30 min, mass of Biochar = 20 mg, and mass of MA@NBAL = 20 mg).

Initial Hg(II) Concentration	Determined Hg(II) Capacity Values (mg/g) at Different Reaction Temperatures (°C)							
	25	30	35	40	45	50	55	60
5 mmol/L	20.6	30.1	40.1	80.2	90.3	90.3	100.3	110.3
10 mmol/L	80.2	120.4	421.2	441.3	441.3	461.4	461.4	481.4

**Table 5:** Computed thermodynamic parameters for Hg(II) adsorption using MA@NBAL at different temperatures.

Temp. (K)	K_d_ (L/g)		Adsorption Thermodynamic Parameters					
			ΔG° (KJ/mol)		ΔS° (J/mol·K)		ΔH° (KJ/mol)	
	5 mmol/L	10 mmol/L	5 mmol/L	10 mmol/L	5 mmol/L	10 mmol/L	5 mmol/L	10 mmol/L
298	0.081	0.011	6.214	11.014	35.392	31.149	16.795	11.466
303	0.088	0.016	6115	10.381				
308	0.095	0.018	6.021	10.153				
313	0.125	0.0194	5.411	10.253				
318	0.132	0.0199	5.335	10.352				
323	0.141	0.0204	5.260	10.449				
328	0.149	0.0209	5.185	10.545				
333	0.157	0.0214	5.110	10.639				

**Table 6:** Comparative performance of MA@NBAL nanobiosorbent versus previously reported biochar and other adsorbents in removal of Hg(II) ions.

Adsorbent	Preparation Method	Optimized Controlling Factor	Maximum Capacity (mg/g)	Application on Real Sample	Ref.
Modified biochar with ferric sulfate		pH = 3.8–7.2 time = 24 h mass = 10 mg T = 20 °C	95.51	It was not applied	[71]
Na_2_S-modified biochar	2 h preparation and pyrolysis at 300 °C	pH = 7.0 time = 12 h mass =150 mg/L T = 25 °C	54.34	It was not applied	[72]
Sulfur-modified pine-needle biochar	Long-duration synthetic approach	pH = 7.0 time = 24 h mass =10 mg T = 5–40 °C	32.0–48.2	It was not applied	[73]
Wood biochar and sulfurized wood biochar	Long-duration synthetic approach	pH = 6.0 time = 0.5–2.0 h mass = 50 mg T = 25 °C	57.8–107.5	It was not applied	[74]
PHPAm/Fe_3_O_4_@SiO_2_-SH	Long-duration synthetic approach	pH = 6.11 time = 115 min mass = 25 mg T = 288–318 K	256.41	It was not applied	[75]
Partially reduced graphene oxide	Long-duration synthetic approach	pH > 4.0 time = 20 min mass = 200 mg T = 298 K	110.21	It was not applied	[76]
Crosslinked hyperbranched polymer modified via sulfhydryl	Long-duration synthetic approach	pH = 4.5 time = 100 min mass = 1.0 g/L T = 318 K.	282.74	It was not applied	[77]
Modified graphene oxide	Long-duration synthetic approach	pH = 5.0 time = 120 min mass = 10 mg T = 273 K	230.0	It was not applied	[78]
MA@NBAL	Rapid and facile microwave-assisted synthesis	pH = 6.0 time = 30.0 min mass = 20 mg T = 20 °C	1444.25	Applied for removal of Hg(II) from wastewater, sea water, and tap water	This study

## Data Availability

The data that support the findings of this study are included in the paper.

## Supplementary Materials

The following supporting information can be downloaded at: https://www.mdpi.com/article/10.3390/nano14221789/s1, Table S1: Specifications of all employed instrumentations with their operational conditions; Table S2: Various kinetic models and definitions of related parameters; Table S3: Various adsorption isotherm models and related parameters; Table S4: Specifications of various water samples; Figure S1: TEM image of MA@NBAL nanobiosorbent; Figure S2: Various kinetic models; Figure S3: Various adsorption isotherm models; Figure S4. Non-linear adsorption isotherm models
