# Supplementary material for: Facile Doping and Functionalization of Molybdic Acid into Nanobiochar to Enhance Mercury Ion Removal from Water Systems
Source: Nanomaterials (Basel). 2024 Nov 7;14(22):1789. doi: 10.3390/nano14221789 (PMC11597330; doi:10.3390/nano14221789)
Supplement: Supplementary file 1 [file nanomaterials-14-01789-s001.zip › nanomaterials-3225153-supplementary.pdf]

Table S1. Specifications of all employed instrumentations with their operational conditions

Characterization	Instrument	Conditions
**SEM**	Scanning electron microscopic JSM-6360LA, JEOL Ltd.	Using an ion sputtering coating device (JEOL-JFC-1100E)
**EDX**	Energy Dispersive X-ray, JSM-IT200, JEOL Ltd	A carbon tap was used as a substrate for EDX measurements. Acceleration voltage 20.00 kV, WD 10.00 mm, Live time 30.00, high vacuum mode
**FT-IR**	BRUKER VERTEX 70 Fourier Transform infrared spectrophotometer	In the range 400–4500 cm^{-1}
**TGA**	CI Electronics microbalance (MK2-MC5)	TGA was acquired under He atmosphere using temperature range 25 – 900°C at 10°C/min ramp
**X-ray photoelectron spectroscopy (XPS)**	Thermo Fisher Scientific (UK) supported instrument with	X-ray source gun A = X-Ray 002 400um - FG ON (400 µm)
**Zeta potential**	Malvern Instruments Ltd	Water dispersant, Dispersant RI is 1.330, Dispersant dielectric constant is 78.5, temperature 25 °C, Zeta potential in mV
**The Brunauer-Emmett-Teller (BET)**	BELSORP-mini II, BEL Japan	The required data were determined by nitrogen adsorption–desorption isotherm measurements, at adsorption temperature 77 K and saturated vapor pressure of 102.48 kPa for 24 h
**UV/ViS Spectrophotometer**	Ultraviolet/visible spectrophotometer by V-530 JASCO	UV/ViS spectrophotometer in between the range of wavelength from 190 nm to 1100 nm was used in the absorption measurement
**Microwave oven**	KOG-1B5H, Korea	Microwave irradiation 1400 W, 2.45 GHz
**pH-meter**	Adwa pH-meter	Standard buffers 4.01, 7.00 and 10.00 were utilized in the calibration of Adwa pH-meter which used in the measurements of solutions pH

Table S2. Various kinetic models and definitions of related parameters.

Kinetic model	Linear form	Parameter definition	Plot
Pseudo-first order	$\ln (q_e - q_t) = \ln q_e - k_1 t$	q_e and qt are the adsorbed amount of Hg(II) (mg/g) at equilibrium and at time t (min), respectively, k_1 is pseudo-first order rate constant (min^{-1}).	Ln (q_e−q_t) vs. the time (t)
Pseudo-second order	$t/q_t = 1/k_2 q_e^2 + t/q_e$	q_e and q_t are the adsorbed amount of Hg(II) (mg/g) at equilibrium and at time t (min), respectively, k_2 stands for the second order rate constant of adsorption (g/(mg min)	t/q_t vs. time (t),
Intra-particle diffusion	$q_t = k_{id} t^{1/2} + C$	k_{id} is the intraparticle diffusion rate constant (mg g^{-1} $min^{-1/2}$). C is the thickness of the boundary layer (mg/g)	(q_t) vs. ($t^{1/2}$)
Elovich	$q_t = \frac{1}{\beta} \ln (\alpha\beta) + \frac{1}{\beta} \ln t$	α is for the initial rate of adsorption (mg g^{-1} min) and β is the activation energy of chemisorption and surface coverage	q_t vs. ln t

Table S3. Various adsorption isotherm models and related parameters.

Adsorption model	Linear form	Parameter definition	Plot
**Langmuir**	$\frac{C_e}{q_e} = \frac{C_e}{q_m} + \frac{1}{q_m b}$<br>$R_L = 1/1+bC_o$ Separation factor:<br>Unfavorable if (R_L>1), favorable (0< R_L<1), linear (R_L = 1) or irreversible (R_L=0)	C_o and C_e is for to the initial and equilibrium concentrations (mg L^{-1}), respectively. qe is the adsorbed phosphate quantity (mg g^{-1}) at equilibrium. q_{max} (mg g^{-1}) and b (L mg^{-1}) are the maximum capacity of adsorption and Langmuir constants	C_e/q_e vs. Ce
**Freundlich**	$\log q_e = \log K_F + \frac{1}{n_f} \log C_e$	q_e is the amount of adsorbed solute and C_e is for the equilibrium solute concentration. K_F (mg g^{-1}) is Freundlich constant, n_f is the intensity of the adsorbents.	log q_e vs. log C_e
**Temkin**	$q_e = (RT/b_T) \ln a_T + (RT/b_T) \ln c_e$<br>$q_e = B \ln a_t + B \ln C_e$<br>$B = \frac{RT}{bt}$	b_T (mg L^{-1}) is the Temkin isotherm constant, a_T (L g^{-1}) is the Temkin isotherm equilibrium binding constant and B is for a constant which expresses the adsorption heat (J/mol)	qe vs. ln Ce
**Dubinin-Radushkevich**	$\ln q_e = \ln q_m - \beta\varepsilon^2$	q_e (mg/g) is the amount of adsorbed heavy metal , qm is the maximum adsorption capacity of adsorbent (mg/g), β is constant related to the adsorption energy.<br>Where Polanyi potential (KJ^2/mol^2) equals:<br>$\varepsilon = RT \ln(1+(1/C_e)$	$\ln q_e$ vs. ε^2

Table S4. Specifications of various water samples**.**

Parameter	Drinking water	Sea water	Wastewater
pH	7.5	7.8	7.9
Na^{+}(mg/L)	18.9	6500	299.4
K^{+}(mg/L)	2.5	440	37.2
Ca^{2+}(mg/L)	25.4	160	114.5
Mg^{2+} (mg/L)	9.3	595	125.1
TDS $^{+}$(mg/L)	185	20750	940.7

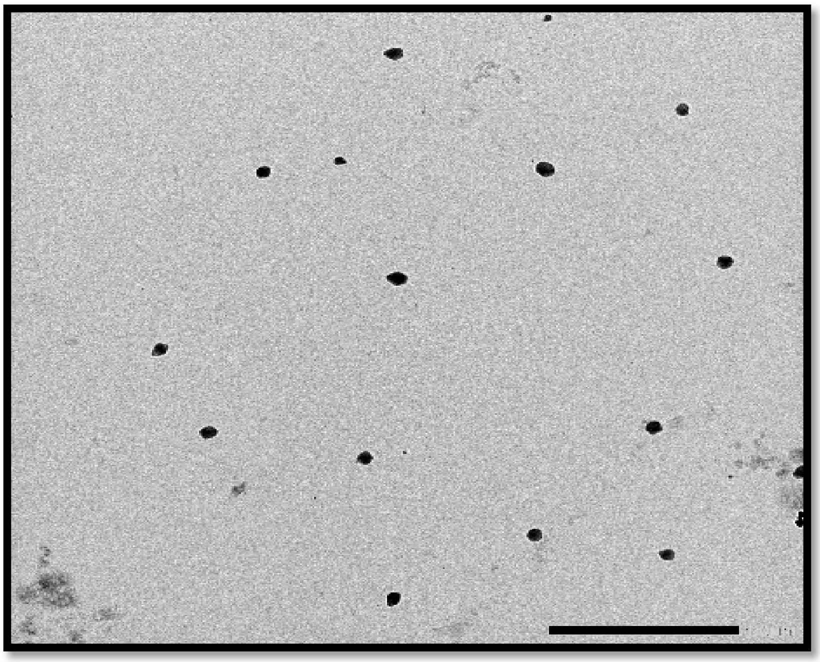

Figure S1. TEM image of MA@NBAL nanobiosorbent

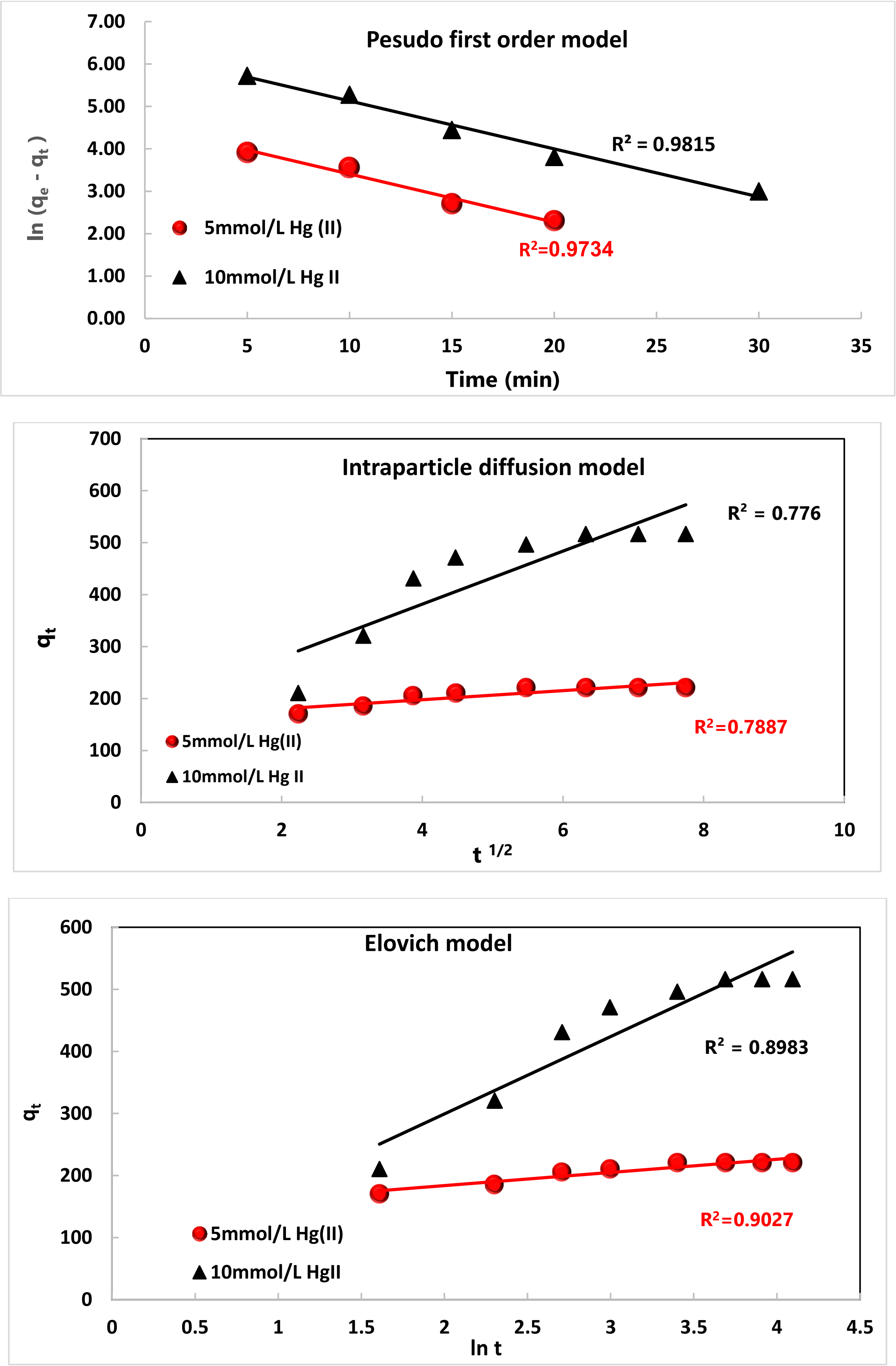

Figure S2. Various kinetic models

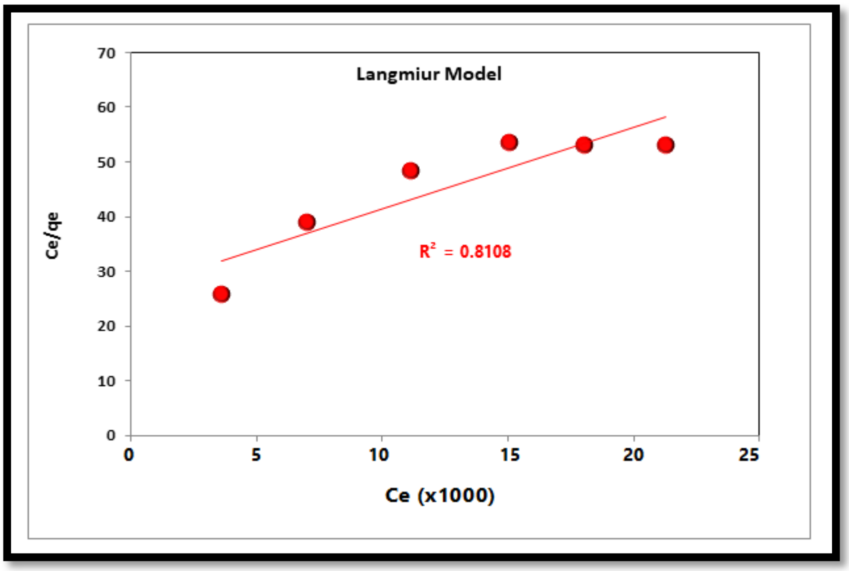

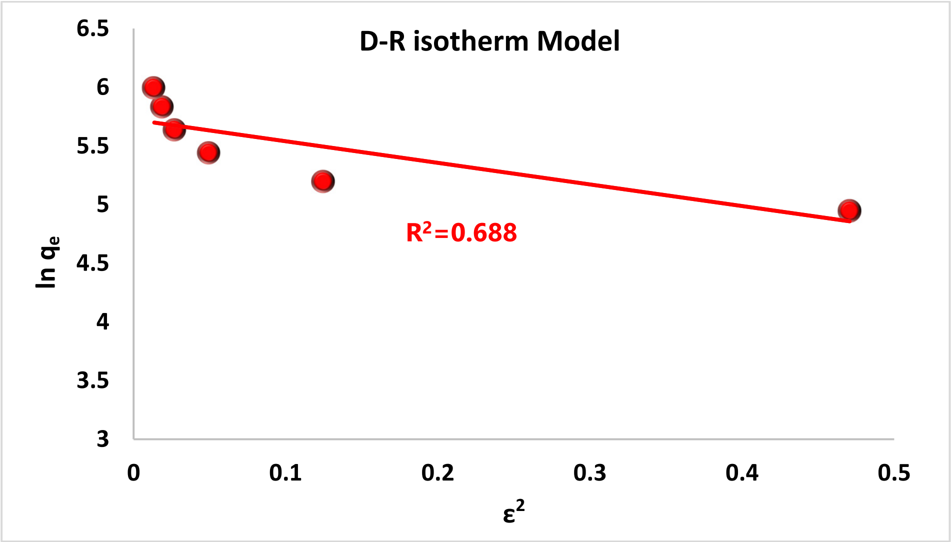

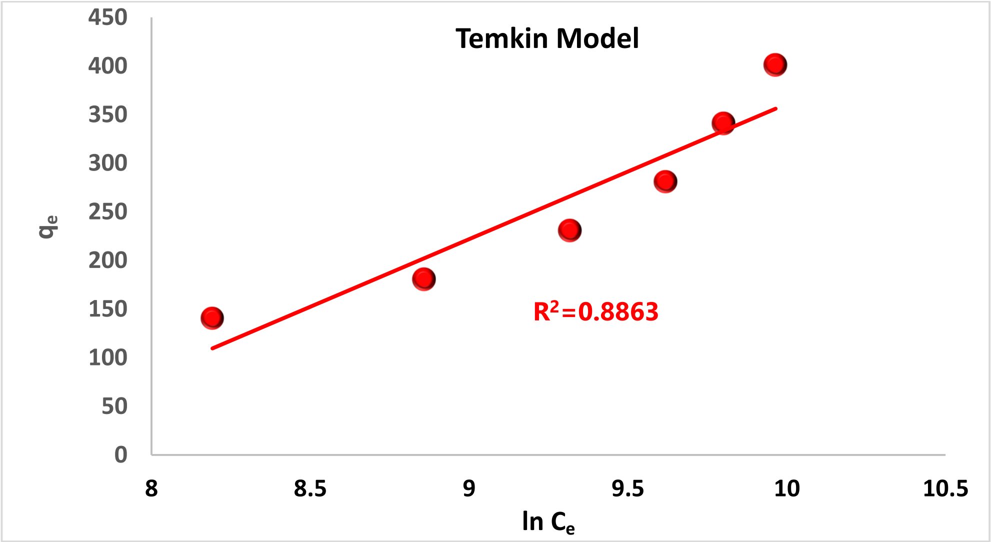

Figure S3. Various linear adsorption isotherm models

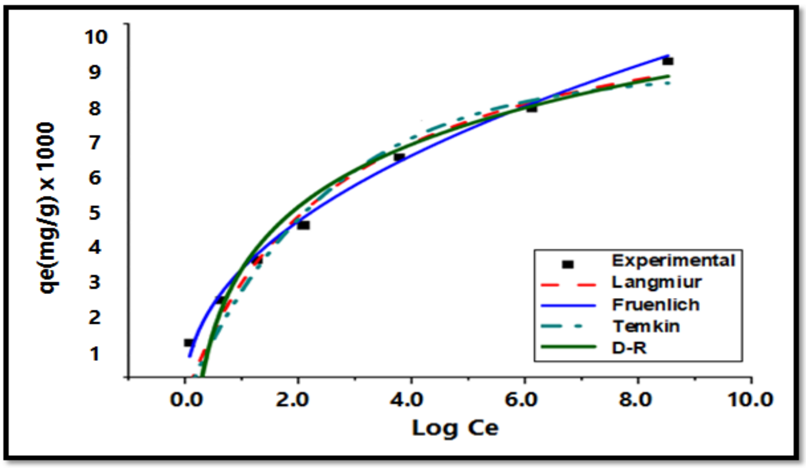

Figure S4. Non-linear adsorption isotherm models
